# Multiplex high-resolution melting assay for simultaneous detection of five key bacterial pathogens in urinary tract infections: A pilot study

**DOI:** 10.3389/fmicb.2022.1049178

**Published:** 2022-12-15

**Authors:** Hossein Kafi, Mohammad Emaneini, Shahnaz Halimi, Hossein Ali Rahdar, Fereshteh Jabalameli, Reza Beigverdi

**Affiliations:** ^1^Department of Microbiology, School of Medicine, Tehran University of Medical Sciences, Tehran, Iran; ^2^Medical Mycology and Bacteriology Research Center, Kerman University of Medical Sciences, Kerman, Iran; ^3^Department of Microbiology, School of Medicine, Iranshahr University of Medical Sciences, Iranshahr, Iran

**Keywords:** urinary tract infections, culture, rapid detection, melting curve, HRM assay

## Abstract

The diagnosis of urinary tract infections (UTIs) is usually based on the results of urine culture, but it is time-consuming, labor-intensive and has a low sensitivity. The aim of this study was to develop multiplex high-resolution melting assay (MHRM) for the simultaneous detection of five common bacterial pathogens (*Escherichia coli*, *Klebsiella pneumoniae*, *Staphylococcus saprophyticus*, *Enterococcus faecalis*, and group B streptococci (GBS)) directly from urine samples. A total of 287 urine specimens were evaluated by HRM assay and the results were compared with the conventional culture method. Five different melt curves generated and differentiated five bacterial pathogens. The detection limit of the MHRM assay was 1.5 × 10^3^ CFU/ml for *E. coli* and *K. pneumoniae* and 1.5 × 10^2^ CFU/ml for *S. saprophyticus*, *E. faecalis* and GBS. Compared to culture, the specificity of the MHRM assay ranged from 99.3 to 100%, and sensitivity 100% for all test pathogens. The MHRM assay developed in the current study might be functional tool for the diagnosis of UTIs and has the potential for direct detection of the organism in the clinical samples. Additionally, it creates results in less than 5 h, helping clinicians to start treatment with appropriate antimicrobial agents. This method could be a useful supplement to urine culture.

## Introduction

Urinary tract infections (UTIs) are considered to be the most common bacterial infections occurring in both the community and hospital setting (Pallett and Hand, 2010). The economic cost of UTIs has been a significant financial burden to patients and health systems (Flores-Mireles et al., 2015). UTIs are most often caused by both Gram-positive and Gram-negative bacteria (Flores-Mireles et al., 2015). The most prevalent causative agent is *Escherichia coli* which responsible for 80–85% of UTIs, followed by *Klebsiella pneumoniae*, *Staphylococcus saprophyticus*, *Enterococcus faecalis*, and group B streptococci (GBS) (Flores-Mireles et al., 2015; Han et al., 2017). In almost all cases of UTIs, empirical antibiotic treatment is initiated before the results of the urine culture are known (Hryniewicz et al., 2001). The widespread use of antibiotics has resulted in the emergence of multidrug-resistant UTI pathogens, resulting in treatment failures (Chen et al., 2013). UTIs are defined as the presence of clinical symptoms (dysuria, pyuria urinary frequency, and urgency) and 10^5^ CFU/ml or greater pathogens in urine (Leber, 2016; Davenport et al., 2017). The gold standard method for diagnosis of UTIs is urine culture, but this is time-consuming, labour-intensive and has a low sensitivity (Jolkkonen et al., 2010; Han et al., 2017). Rapid, simple and accurate methods for the detection of UTI pathogens are essential for the selection of antibiotics and patient management (Raja et al., 2017). Various approved methods have been developed to improve the diagnosis of UTIs (Davenport et al., 2017; Santos et al., 2022). Urine dipstick tests for urine nitrite and leukocyte esterase are widely used for early diagnosis of UTI, because of their cost effectiveness and clinical utility. But, they do not identify the causative bacteria and also have poor sensitivity (Davenport et al., 2017; Kapur et al., 2019). Lateral flow assays including RapidBac have also been developed for UTI diagnosis. These assays are rapid and inexpensive; but, have poor specificity and sensitivity (Davenport et al., 2017). VITEK 2 microbial identification system can identify bacteria from primary cultures of urine in about 5 h, but it is costly and requires single colony from pure cultures of the microorganism. Matrix-assisted laser desorption ionization–time of flight mass spectrometry (MALDI-TOF MS) is another tool used for early identification of bacteria in urine, blood cultures, respiratory tract secretions, cerebrospinal fluids and stool samples (Singhal et al., 2015). It is a quick and reliable technology for the identification of microorganisms, but the cost of the instrument is high and it requires trained personal (Davenport et al., 2017). Fluorescence *in situ* hybridization and multiplex PCR (GeneXpert and Cepheid) have been used for the diagnosis of UTIs (Davenport et al., 2017). They are specific, sensitive, and rapid, but they require multiple specific probes for different uropathogens and extensive sample preparation. Forward light scattering systems like BacterioScan and Uro-Quick can provide results within 45 min, but they are not able to identify the pathogen (Davenport et al., 2017). Recently, high-resolution melting (HRM) has received more attention as a means of detecting of nosocomial pathogens, bacterial resistance genes, genotyping, detection and differentiation of various pathogenic organisms (Bender et al., 2020; Xiu et al., 2020; Girault et al., 2022; Pakbin et al., 2022; Perini et al., 2022). This method relies on the accurate monitoring of the change in fluorescence of intercalating dyes as the double stranded DNA (dsDNA) melts following amplification in the presence of a saturating DNA intercalating dye (Silva et al., 2022). The melting temperature (Tm) is the point at which half of total quantity of dsDNA has separated and differences in the Tm profile allow us to identify and discriminate various infectious agents (Chua et al., 2015; Silva et al., 2022). However, this technique has not been applied directly on urine samples to simultaneously differentiate several pathogens. The aim of this study was to develop a HRM-based assay for the simultaneous detection of five common bacterial pathogens (*E*. *coli*, *K*. *pneumoniae, S*. *saprophyticus*, *E*. *faecalis*, and GBS) directly from urine samples.

## Materials and methods

A total of 287 mid-stream urine specimens were collected from patients (out patients and in patients) in Imam Khomeini hospital, Tehran-Iran, between August 2019 and October 2019. Each urine specimen had an approximate volume of 15–20 ml, which was split into two aliquots. The first aliquot was used for quantitative urine culture and the second aliquot was transferred into a 1.5 ml sterile microcentrifuge tube and frozen at −80°C until the DNA extraction. All urine specimens were inoculated on blood agar and MacConkey agar (Conda Pronadisa, Spain) with a calibrated 0.01-ml disposable loop. After at least 24 h incubation at 37°C, the colony counts were carried out and recorded. The isolation of three or more species in a urine culture usually considered as contaminants. Bacterial colony counts and species identification were performed based on validated standard operation procedures (Leber, 2016). Urine cultures were considered positive if 10^5^ bacteria or more per milliliter of urine were found (Leber, 2016).

### DNA extraction

DNA was directly extracted from 200 μl of urine that was stored at −80 freezers using the Favorgen DNA extraction Kit (Biotech Corp, Taiwan). The concentration and the purity of all DNA were determined with a NanoDrop 2000c instrument (Thermo Fisher Scientific, United States). All DNA preparations were stored at 4°C until used.

### Primer design

The whole genome sequences of *E*.*coli* (GenBank accession number NC_017626.1), *K*. *pneumoniae* (GenBank accession number NC_022566), *S*. *saprophyticus* (GenBank accession number CP075502.1), *E*. *faecalis* (GenBank accession number NC_017312.1), and GBS (GenBank accession number NC_019048) were downloaded from NCBI database. Comparative analysis of the chromosomes of these five species was carried out for identifying conserved regions (species-specific sequences). Five primer sets were designed using an online Primer3 software^1^ and the specificity of primers was evaluated *in silico* using primer-BLAST program available at.^2^ The theoretical melting temperature (Tm) of each amplicon was determined with OligoCalc (Oligonucleotide Properties Calculator) based on the amplicon sequence.^3^ The five primer sets produced 97–272 bp products and the sequences of the forward and reverse primer, their targets, and predicted amplicon Tm are described in Table 1. The efficiency of primers was assessed by using conventional PCR. Initially, the genomic DNA of five different species were used as templates to determine the optimum annealing temperatures in separate PCR reactions (one primer set in each tube). To perform and optimize the multiplex reaction, we then included all five primer sets in each tube to ensure that primers were able to amplify their own target region in the five species without creating unspecific products or primer dimers that could interfere with the interpretation of results in PCR-HRM analysis later on. The reaction mixture contained 12.5 μl PCR Master Mix 2X (Ampliqon, Denmark), 0.5 μl of each primer (10 pmol, Metabion, Martinsried, Germany), 1 μl of DNA (10–20 ng/μl) and 6.5 μl of DNase-free water in a total reaction volume of 25 μl per sample. The amplification conditions were 5 min at 95°C, followed by 30 cycles of 95°C for 45 s, 63°C for 20 s and 72°C × 25 s and a final extension at 72°C for 5 min. The reactions were performed in a T100™ thermal cycler (Bio-Rad). The amplified DNA fragments were electrophoresed in a 1.5% agarose gels with 0.5X TBE (Tris/Borate/EDTA) buffer. The DNA bands were visualized by KBC power load dye staining and photographed under UV illumination.

**Table 1:** List of primers used in this study and its properties.

Pathogen	Gene Bank accession no.	primer sequence/Tm (°C)	Nucleotide positions	Amplicon size (bp)	GC content of amplico*n* (%)	Predicted Tm^a^ (°C)	Observed Tm^b^ (°C)
*E. coli*	NC_017626.1	F: CATACCTGTTCACCGACGAC (55.4)	1,863,749–1,863,922	174	53	84.59	87.73
		R: CTGGCAGGAGAAACTGCATC (56.1)					
*K. pneumoniae*	NC_022566	F: GGCGAGGTTTACGTCTCAAC (55.9)	4,708,186–4,708,457	272	61	87.59	90.51
		R: GTACTTCTTGTTGGCCTCGC (56.2)					
*E. faecalis*	NC_017312.1	F: TGTTGTATGGCGGCAGAAGT (58)	1,198,949–1,199,147	199	36	79.07	80.99
		R: TCAGGTGTTTGTGCCCAAGT (58)					
*S. saprophyticus*	CP075502.1	F: TTCTAGTCATTACGTCGCTCCT (60)	2,445,607–2,445,717	111	28	73.28	77.02
		R: AGTTTTACACTTTTGGGAAGCGT (59)					
GBS	NC_019048	F: CTG TAAAATCTCAACGTGGACG (60)	1,008,163–1,008,259	97	35	75.36	79.1
		R: GCATGACTTTCAAAGACTGAGAG (59)					

^a^Amplicon melt point calculated by OligoCalc program. ^b^Amplicon melt point calculated by ABI StepOnePlus Real-Time PCR instrument.

### Multiplex HRM-real-time PCR assay

Multiplex real-time PCR with HRM (MHRM) were done sequentially on a ABI StepOnePlus Real-Time PCR (Applied Biosystems) in a reaction mix containing 4 μl of 5x HOT FIREPol^®^ EvaGreen^®^ HRM Mix no ROX (Soils Biodyne, Estonia), 0.5 μl of each primer (10 pmol, Metabion, Martinsried, Germany), 1 μl of DNA (10–20 ng/μl) and 6.5 μl of DNase-free water in a total reaction volume of 20 μl per sample. Positive controls (genomic DNA from each species) and negative controls (distilled water) were used in each run. The reaction conditions were enzyme activation at 95°C for 15 min, followed by 40 cycles of denaturation at 95°C for15 s, 63°C for 20 s for annealing and 72°C for 20 s for extension. Following this, HRM was performed at 95°C for 15 s and a melting profile from 65°C to 95°C using a ramping degree of 0.3°C/s. Then, the melt curve analysis was performed by HRM Software version 3.0.1 (Applied Biosystems). All samples were analyzed at least in triplicate.

The specificity of the HRM assay was evaluated using different DNA from other organisms such as; bacterial DNA of *S*. *aureus*, *E. faecalis*, *Streptococcus pyogenes*, *Proteus mirabilis* and *Salmonella* Typhimurium. Moreover, DNA from urogenital flora including *Lactobacillus* spp., *S. epidermidis*, yeast, viridans and nonhemolytic streptococci was also included in the assay.

### Limit of detection

The limit of detection (LOD) of HRM assay was determined using serial 10-fold dilutions of 0.5 McFarland suspension of each isolate, ranging from 10^7^ to 10^1^ CFU/ml. A 10 μl sample of each dilution was plated and the colonies were counted to determine the CFU/ml in each dilution. Genomic DNA was extracted from all dilutions by DNA Extraction Kit (Biotech Corp, Taiwan), according to the manufacturer’s instructions. We calculated the sensitivity and specificity of the real-time PCR with HRM analysis for the detection of five common bacterial pathogens compared with urine culture, which was used as the reference standard. The sensitivity of this assay was calculated as: 1 − [true positive /(true positive + false negative)] × 100%, while the specificity of this assay was calculated as 1 − [true negative/(false positive + true negative)] × 100%. True positive and true negative were defined as culture- positive and culture- negative urine sample, respectively. The HRM positive, culture- negative and the HRM negative, culture- positive were defined as false positive and false negative, respectively.

## Results

### Multiplex HRM-PCR can detect and differentiate common pathogen in urine

**Figure 1:**
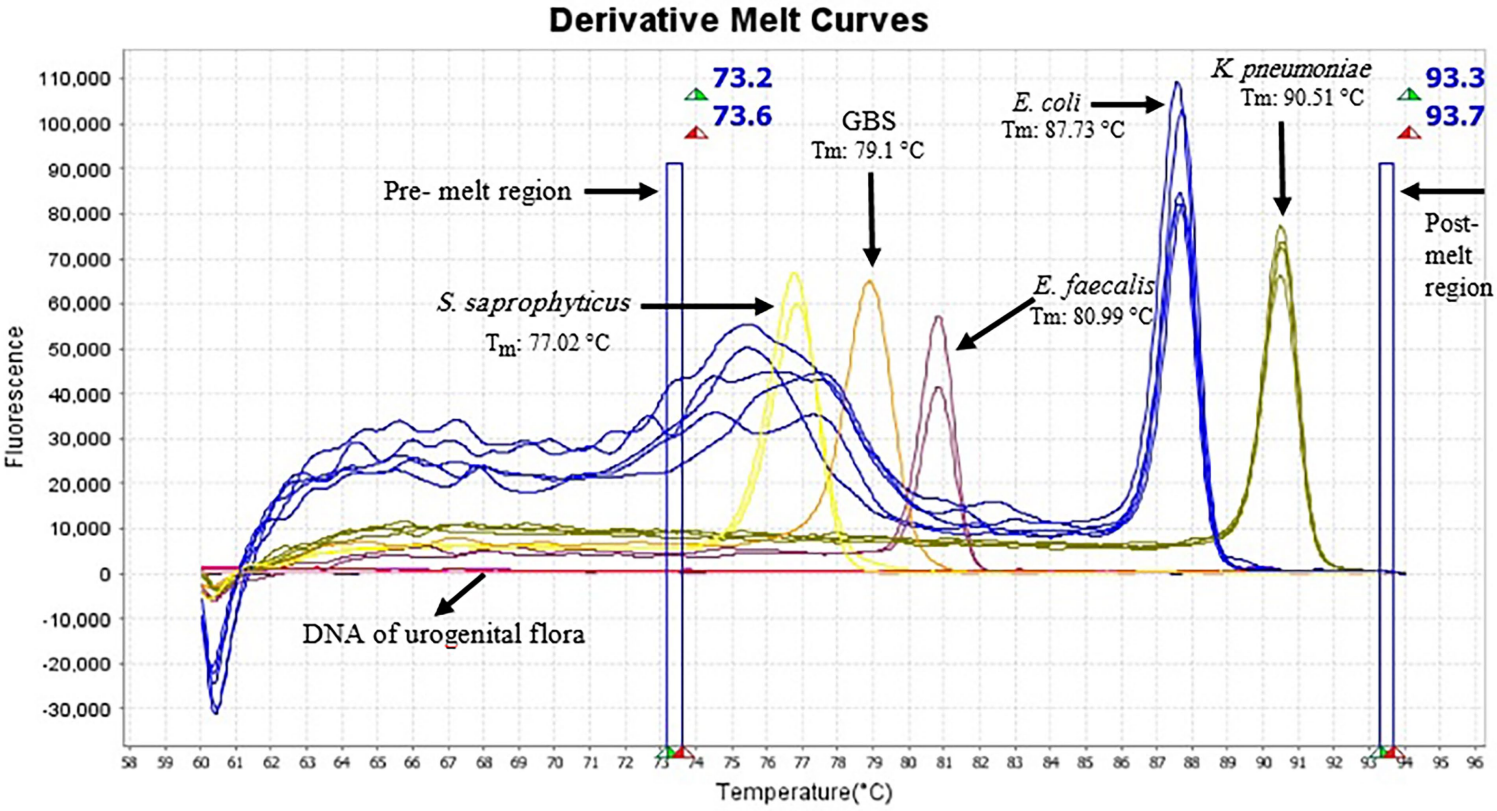
Derivative melting curves of HRM analysis of five different species generated from urine samples, each displaying a unique profile with respect to shape and melting temperature. Each line represents one sample and the same colors illustrate the same pathogens. Yellow curve (*S. saprophyticus*, *n* = 2); light orange (GBS, *n* = 1); plum (*E. faecalis*, *n* = 2); blue (*E. coli*, *n* = 5) and dark yellow (*K. pneumoniae*, *n* = 4). Pre-melt region: The set of lines to the left of the peak displays the pre-melt start and stop temperatures when every amplicon is double-stranded. Post-melt region: The set of lines to the right of the peak displays the post-melt start and stop temperatures when every amplicon is single-stranded. The HRM analysis was performed with a pre-melt region 73.2–73.6°C and a post-melt region 93.3–93.7°C. No amplification occurred in DNA samples of urogenital flora and therefore no melting curve was observed.

**Figure 2:**
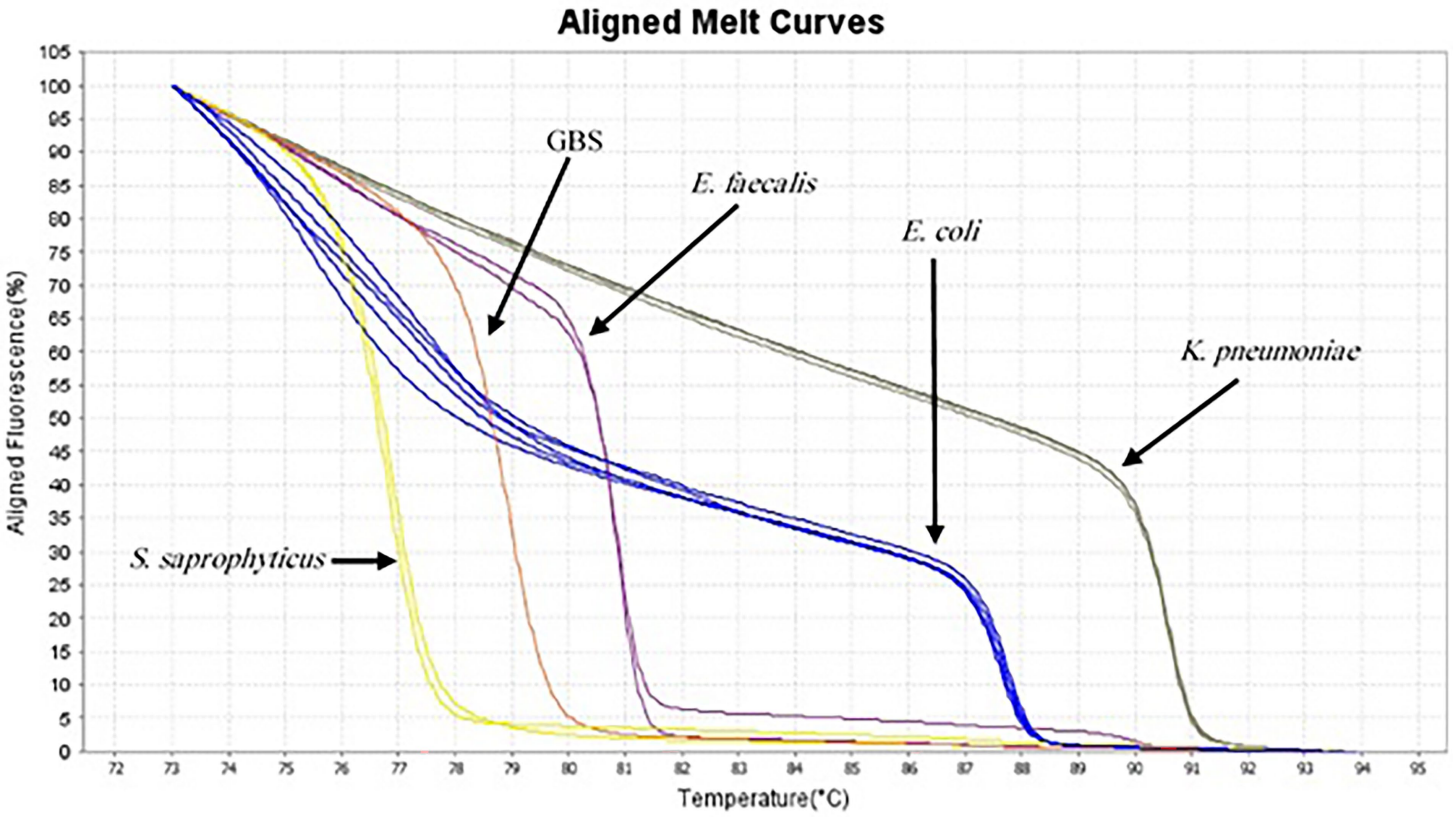
Aligned melt curves of five different species generated from urine samples (as described in the legend to Figure 1) from the lowest Tm to highest.

AS described in the Methods section, the multiplex HRM assay was designed to simultaneously detect and differentiate five common bacterial pathogens (*E. coli*, *K. pneumoniae*, *S. saprophyticus*, *E. faecalis* and GBS) directly from urine samples. The patterns of the derivative melting curve and aligned melt curve are shown in Figures 1, 2, respectively. As shown in the plots, each species shown a unique Tm that helped to differentiate the tested bacteria at the species level in a single HRM reaction. The melting curves were characterized by peaks of 77.02 ± 0.3°C for *S*. *saprophyticus*, 79.1 ± 0.4°C for GBS, 80.99 ± 0.2°C for *E*. *faecalis*, 87.73 ± 0.5°C for *E*. *coli*, 90.51 ± 0.4°C for *K*. *pneumoniae*.

### Comparison of HRM assay to culture in identification of pathogens

In total, 287 mid-stream urine samples were analyzed, of those 223 specimens (77.7%) were negative for both culture and HRM. Sixty- one samples (21.2%) were culture positive (CFU > 10^5^) and 64 samples (22.3%) were positive in HRM. All culture-positive samples were also HRM positive, with detailed species identification (Table 2). *E*. *coli* (14.3%, 41/287) was the most common pathogen by both culture and HRM assay. The isolation of other pathogens was presented in Table 2. In 3 of 64 HRM positive samples, two samples with two melting peaks were observed in the HRM derivative plots, suggesting the presence of two pathogens (one samples: *E*. *coli* + *E*. *faecalis* and one sample: *E*. *coli* + GBS) but culture identified one pathogen (*E*. *coli*) in two samples (Table 2) (double bacterial melt curves are shown in Supplementary Figures S1, S2). One sample was HRM positive (*E*. *faecalis*) but negative by culture.

**Table 2:** Comparative analysis of HRM and culture identification.

No	HRM positive for organism(s)	Culture positive for organism(s)	Quantity CFU/ml
39	*E. coli*	*E. coli*	>10^5^
11	*K. pneumonia*	*K. pneumoniae*	>10^5^
5	*E. faecalis*	*E. faecalis*	>10^5^
4	*S. saprophyticus*	*S. saprophyticus*	>10^5^
2	GBS	GBS	>10^5^
1	*E. coli*, *E. faecalis*	*E. coli*	>10^5^
1	*E. coli*, GBS	*E. coli*	>10^5^
1	*E. faecalis*	—	—

Quantities apply only to cultures.

### LOD, specificity and sensitivity of HRM assay

The LOD of HRM assay for *E*. *coli* and *K. pneumoniae* was 1.5 × 10^3^ CFU/ml, and for *S*. *saprophyticus, E*. *faecalis* and GBS was 1.5 × 10^2^ CFU/ml. Compared to culture, the specificity of the HRM assay ranged from 99.3 to 100%, and sensitivity 100% for all test pathogens (Table 3).

**Table 3:** Comparison of sensitivity and specificity of HRM assay versus culture identification method.

Target	True positive	True negative	False positive	False negative	Sensitivity (%)	Specificity (%)
*E. coli*	41	246	0	0	100	100
*K. pneumonia*	11	276	0	0	100	100
*E. faecalis*	5	280	2	0	100	99.3
*S. saprophyticus*	4	283	0	0	100	100
GBS	2	284	1	0	100	99.6

## Discussion

Early diagnosis of UTI infections is vital to effective treatment, especially in developing countries such as Iran where the misuse and overuse of antibiotics are very common, leading to the emergence of resistant strains and subsequent treatment failures (Raja et al., 2017; Lee et al., 2018; Beigverdi et al., 2019). In recent years, molecular assays have opened new avenues for diagnosis of infectious disease (Raja et al., 2017). However, reports describing MHRM assay for the diagnosis of bacterial UTIs have not been described. To our knowledge, this is the first report of a single-tube HRM assay for the simultaneous detection and accurate differentiation of five major bacterial pathogens directly from urine samples. Our assay was highly specific for the targeted organism as none other organism than the five tested bacteria could be amplified, and no detectable change in the fluorescence signal was detected in NTCs as well. Different amplicons containing regions with different GC content, sequence, length and difference in Tm values easily differentiated all 5 different species based on changes in the melting curve profile (Khosravi et al., 2017; Edwards et al., 2018; Xiu et al., 2020; Pakbin et al., 2022). In our study, the melting profile of unknown samples was correctly matched with melting profiles of standards with known DNA. Despite its discriminatory power, this assay can provide results within less than 5 h in comparison with biochemical or culture based methods, for which results are available after 24–48 h. Another advantage of this assay is its low cost, because it eliminates the need for expensive fluorescent-labeled probes and do not need gel electrophoresis and sequencing of the products (Zampieri et al., 2016; Gelaye et al., 2017; Khosravi et al., 2017). The cost of this assay from sample preparation, DNA extraction and HRM run was about $3 per test, but this technique requires expensive equipment not available everywhere. Sun et al. reported that the average cost of HRM assay was $2 per sample for detecting of four resistance genes in single reaction (Sun et al., 2019). Quantification of the bacteria in the UTIs is the critical to differentiate between contamination and infection (Franz and Hörl, 1999; Leber, 2016). In current study, the LOD of the HRM assay ranged between 1.5 × 10^3^ CFU/ml for *E*. *coli* and *K. pneumoniae* and 1.5 × 10^2^ CFU/ml for *S*. *saprophyticus, E*. *faecalis* and GBS, which is lower than the number of bacteria suggested for identification of bacterial infection *via* culture (10^5^ CFU/ml). Therefore, samples which are reported as culture negative and HRM positive cases can be explained as contamination or colonization. In our study, three false positive results were obtained with the HRM method. In our study, three false positive results were obtained with the HRM method. For 2 samples, culture identified one organism (*E. coli*), while HRM identified two organisms (*E. coli* + *E. faecalis* and *E. coli* + GBS). In another sample, culture was negative but HRM identified one organism (*E. faecalis*). False positive results could be attributed to the amplification of DNA from dead bacteria present in the urine, low concentrations of the organisms, presence of viable but not culturable bacteria, prior antimicrobial therapy or sampling error. In this study, the HRM assay revealed more than 99% specificity and 100% sensitivity for each pathogen. Similar findings were observed in the study of Edwards et al. in which HRM assay was used for the identification of six common Gram-negative pathogens and their data showed that the sensitivity and specificity of HRM assay were 97.1 and 100%, respectively. It should be noted that a major drawback of HRM approach is the lack of ability to differentiate the closely related species (Yang et al., 2009). One of the potential applications of HRM assay in detecting UTIs is to detect the severity of the infection(s). To this end one needs to compare the cycle threshold (CT) for different positive samples and use these values to determine the load of the pathogen in unit volume of urine. In this study we did not examine the severity of the infection, but it remains to be studied in the future. Moreover, to be used in clinical settings, the developed assay needs to be further evaluated with a higher sample size, *via* double blinded study followed by rigorous statistical analyses.

## Conclusion

The MHRM assay developed in current study seems to be a promising tool for simultaneous detection and identification of common bacterial pathogens directly from urine samples. Additionally, it provides results in less than 5 h, helping clinicians to start treatment with appropriate antimicrobial agents. This method could be a useful supplement to urine culture.

## Data availability statement

The raw data supporting the conclusions of this article will be made available by the authors, without undue reservation.

## Ethics statement

The studies involving human participants were reviewed and approved by the study protocol was conducted according to the recommendations of the Ethics Committee of Tehran University of Medical Sciences (ethical approval reference number IR.TUMS.MEDICINE.REC.1397.457) in compliance with the Ethical principles of the Declaration of Helsinki on medical research involving human subjects. The patients/participants provided their written informed consent to participate in this study.

## Author contributions

RB designed the experiments. HK conducted the experiments. SH and HR contributed materials and analysis tools. RB and FJ drafted the manuscript. ME revised the manuscript and advised in all parts of the study. All authors have read and approved the final manuscript.

## Funding

This research has been supported by Tehran University of Medical Sciences and Health Services grant 97-02-30/38781.

## Conflict of interest

The authors declare that the research was conducted in the absence of any commercial or financial relationships that could be construed as a potential conflict of interest.

## Publisher’s note

All claims expressed in this article are solely those of the authors and do not necessarily represent those of their affiliated organizations, or those of the publisher, the editors and the reviewers. Any product that may be evaluated in this article, or claim that may be made by its manufacturer, is not guaranteed or endorsed by the publisher.

## Supplementary material

The Supplementary material for this article can be found online at: https://www.frontiersin.org/articles/10.3389/fmicb.2022.1049178/full#supplementary-material

## Footnotes

^1^ http://primer3.ut.ee/

^2^ https://www.ncbi.nlm.nih.gov/tools/primer-blast/

^3^ http://biotools.nubic.northwestern.edu/OligoCalc.html
