# Supplementary figures and images for: Multiplex high-resolution melting assay for simultaneous detection of five key bacterial pathogens in urinary tract infections: A pilot study
Source: Front Microbiol. 2022 Dec 15;13:1049178. doi: 10.3389/fmicb.2022.1049178 (PMC9797728; doi:10.3389/fmicb.2022.1049178)

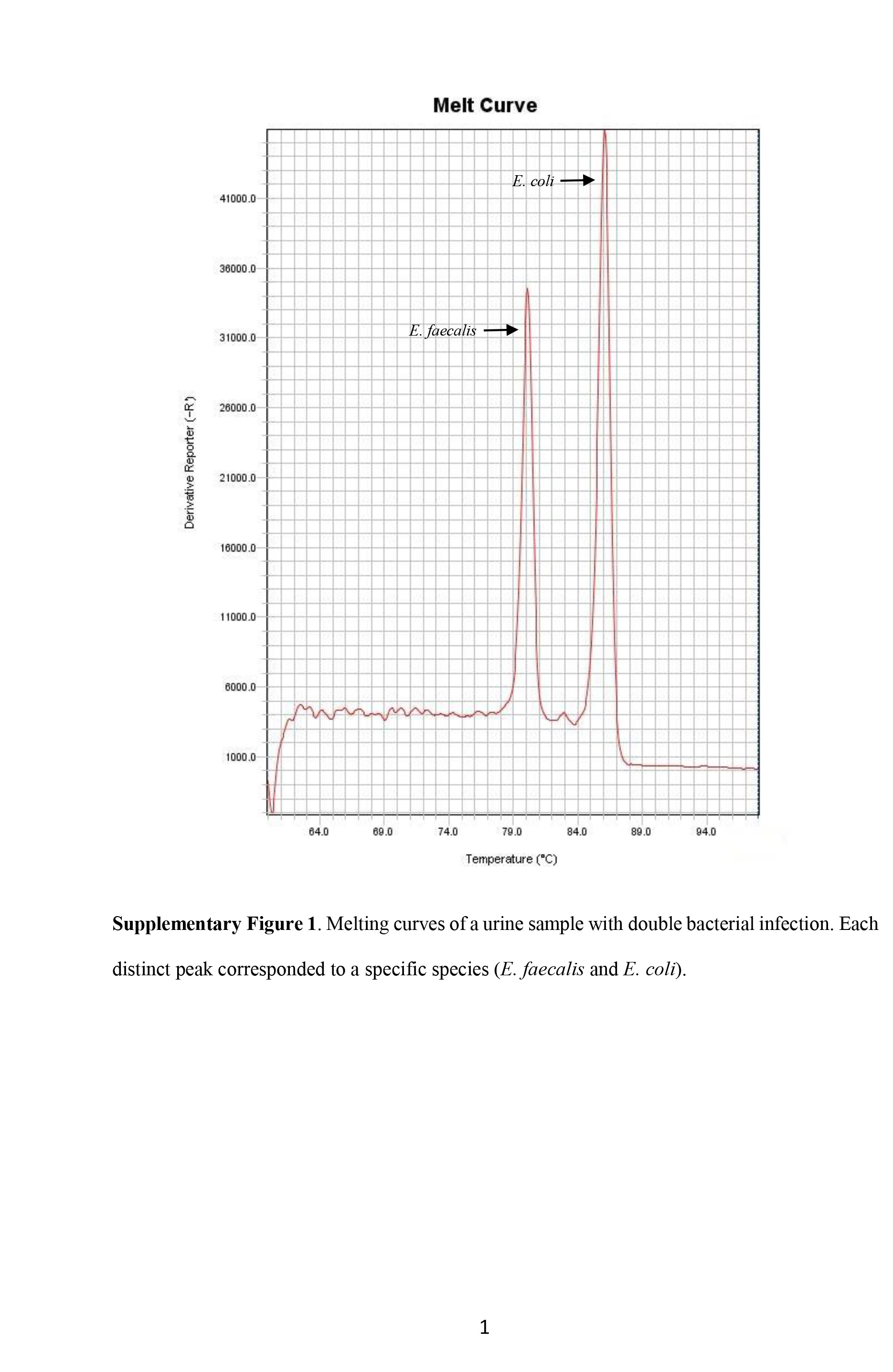

Supplement: Supplementary file 1 [file Image_1.jpg]

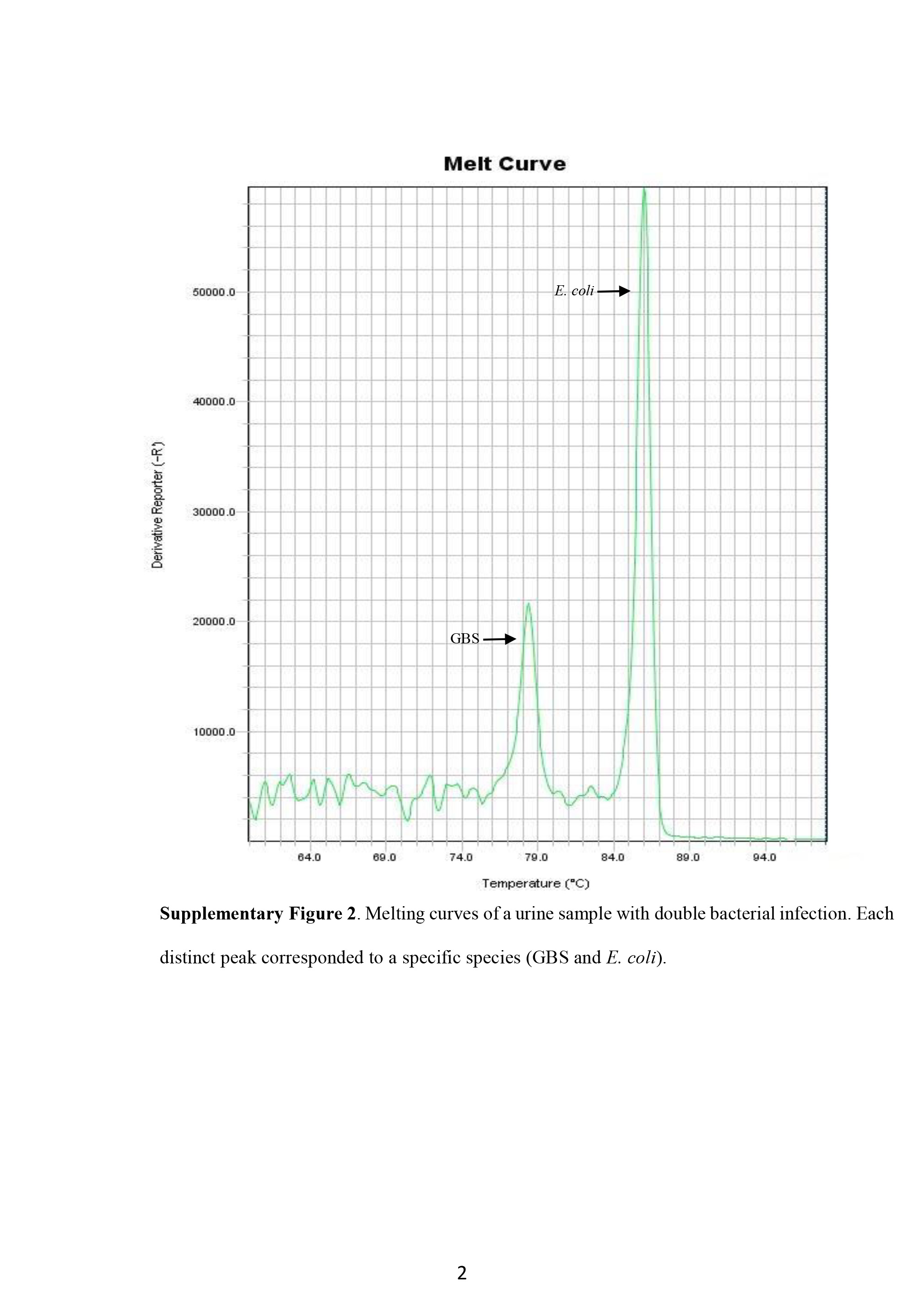

Supplement: Supplementary file 2 [file Image_2.jpg]
